# Enhancement of Tomato Fruit Quality Through Moderate Water Deficit

**DOI:** 10.3390/foods13223540

**Published:** 2024-11-06

**Authors:** Yongmei He, Junwen Wang, Jiaojiao Yang, Peng Bai, Junfang Feng, Yue Wu, Jihua Yu, Linli Hu, Weibiao Liao

**Affiliations:** 1 College of Horticulture, Gansu Agricultural University, Lanzhou 730070, China; 17339904893@163.com (Y.H.); wangjw0314@163.com (J.W.); yjj19990703@163.com (J.Y.); baipengbaipeng666@163.com (P.B.); 17752271685@163.com (J.F.); yujihua@gsau.edu.cn (J.Y.); hull@gsau.edu.cn (L.H.); liaowb@gsau.edu.cn (W.L.); 2 State Key Laboratory of Aridland Crop Science, Gansu Agricultural University, Lanzhou 730070, China

**Keywords:** irrigation management, tomato, growth physiology, fruit development, fruit characteristics, nutritional quality, flavor quality

## Abstract

In arid areas, water shortage has become a major bottleneck limiting the sustainable development of agriculture, necessitating improved water use efficiency and the full development of innovative water-saving irrigation management technologies to improve quality. In the present study, tomato (*Solanum lycopersicum* cv. Micro Tom) fruits were used as materials, and different irrigation frequencies were set during the fruit expansion stage. The normal treatment (CK) was irrigated every three days, while the water deficit treatments were irrigated at varying frequencies: once every 4 days (T1), 5 days (T2), 6 days (T3), 7 days (T4), and 8 days (T5). These corresponded to 80%, 70%, 60%, 50%, and 40% of the maximum field moisture capacity (FMC), respectively, with CK maintaining full irrigation at 90% of the maximum FMC. The water deficit treatment T3, with less stress damage to plants and the most significant effect on fruit quality improvement, was selected based on plant growth indices, photosynthetic characteristics, chlorophyll fluorescence parameters, and fruit quality indices, and its effects on carotenoids, glycolic acid fractions, and volatile compounds during tomato fruit ripening were further investigated. The outcome indicated that moderate water deficit significantly increased the carotenoid components of the tomato fruits, and their lycopene, lutein, α-carotene, and β-carotene contents increased by 11.85%, 12.28%, 20.87%, and 63.89%, respectively, compared with the control fruits at the ripening stage. The contents of glucose and fructose increased with the development and ripening of the tomato fruits, and reached their maximum at the ripening stage. Compared to the control treatment, the moderate water deficit treatment significantly increased the glucose and fructose levels during ripening by 86.70% and 19.83%, respectively. Compared to the control conditions, water deficit conditions reduced the sucrose content in the tomato fruits by 27.14%, 18.03%, and 18.42% at the mature green, turning, and ripening stages, respectively. The moderate water deficit treatment significantly increased the contents of tartaric acid, malic acid, shikimic acid, alpha ketoglutaric acid, succinic acid, and ascorbic acid, and decreased the contents of oxalic acid and citric acid compared to the control. The contents of total soluble sugar and total organic acid and the sugar–acid ratio were significantly increased by 48.69%, 3.71%, and 43.09%, respectively, compared with the control at the ripening stage. The moderate water deficit treatment increased the fruit response values to each sensor of the electronic nose, especially W5S, which was increased by 28.40% compared to the control at the ripening stage. In conclusion, during the ripening process of tomato fruit, its nutritional quality and flavor quality contents can be significantly improved under moderate (MD) deficit irrigation treatment. The results of this experiment can lay the foundation for the research on the mechanism of water deficit aiming to promote the quality of tomato fruit, and, at the same time, provide a theoretical basis and reference for tomato water conservation and high-quality cultivation.

## 1. Introduction

Water is the primary environmental factor influencing plant growth, development, and the formation of quality and yield. However, it is anticipated that, due to global climate change, extreme weather events such as droughts will intensify in frequency, severity, and geographical extent, leading to a reduction in available water resources [1]. Therefore, improving the efficiency of water resource utilization has become an important issue in agricultural development. Traditional irrigation theory posits that any level of water deficit will invariably diminish crop yield and quality. However, numerous researchers and scholars have observed that, while water deficit can adversely affect crop growth, development, and eventual yields, moderate water deficit can actually enhance crop quality [2,3]. For example, moderate water deficit could increase the oil content of soybean [4]. Deficit irrigation could increase the total soluble solids, reducing sugars, organic acids, vitamin C, and lycopene content of tomato fruits [5]. Researchers found that deficit irrigation led to a decrease in tomato plant growth and fruit yield, size, and number. However, it resulted in enhanced fruit color, reduced water content, and increased concentrations of sucrose, glucose, and fructose compared to well-watered crops [6]. Therefore, in irrigation management for vegetable production, it is imperative to consider not only yield but also its quality.

Tomato (*Solanum lycopersicum*) belongs to the *Solanaceae* family and is an important vegetable crop [7]. Native to Central and South America, it is favored for its nutrients such as its vitamins, organic acids, lycopene, and carbohydrates [8]. According to the Food and Agriculture Organization of the United Nations database, the global production of tomato in 2022 was 2.54 × 10^12^ kg, and China’s production was 1.37 × 10^12^ kg, with China accounting for 53.72% of global production (http://www.fao.org/faostat/en/#home). Tomatoes are extensively utilized and highly sought after in the global market, ranking among the most popular vegetables worldwide. Particularly, the global demand for tomatoes and their processed derivatives has surged significantly in recent years, leading to a growing share of tomatoes in the vegetable trade [8]. With the increasing demand of consumers for delicious, nutritious, and high-quality vegetables, and the desire of growers and the industry to gain more profits, the relevant research on tomato quality improvement has attracted more and more attention [9,10]. It has been found that soil moisture significantly regulates both the growth and quality of tomato, and that either too high or too low a level of irrigation can inhibit the growth and development process and quality formation [11]. Deficit-regulated irrigation represents an innovative ecological approach to water conservation and cultivation. This method not only curtails water usage and enhances water utilization efficiency but also fosters plant root growth and facilitates improved distribution of dry matter. Consequently, it contributes to enhancing the quality and flavor of the crop [7,12]. To achieve high-quality tomatoes while maintaining satisfactory yields, it is crucial to develop an irrigation program that balances maximizing tomato yield with optimizing fruit quality.

The sustainable use of water resources for agriculture is a key challenge. Adopting water-efficient irrigation techniques while maintaining good harvests can help conserve this increasingly scarce vital resource [13]. In areas with water shortages and long-term droughts, prioritizing increasing the efficiency of water use rather than maximizing crop output may better serve farmers [14]. Some studies have reported that drought stress during the nutrient period does not adversely affect the yield of tomato [3,15,16]. It has been found that drought stress applied at the fruit-ripening stage tends to improve fruit quality traits such as fruit firmness, color index, total soluble solids, vitamin C, soluble sugars, and organic acids in tomatoes, despite the negative effects on tomato fruit yield [17]. Abiotic stress such as that caused by drought, alkali, or salt increases the amount of ethylene in tomatoes, which, in turn, raises carotenoid and lycopene concentrations, enhancing the color of the tomato skin [18,19]. Under the stress of water deficit, the increase in soluble solids in tomatoes was attributed to the reduced water intake and the maintenance of organic solute synthesis and accumulation [20]. It has also been shown that reducing irrigation improved quality in terms of soluble solids, ascorbic acid, acidity, and color index (lycopene) but affected fruit size [21]. Although numerous studies have investigated the effects of water deficit irrigation on crop growth and quality, the treatment method was basically to reduce the corresponding field water capacity. In this experiment, the same irrigation volume is applied to tomato plants at different irrigation frequencies. This approach is simpler, less time consuming, and labor efficient.

In this study, the tomato (*Solanum lycopersicum* cv. Micro Tom) is regarded as the material to research the impacts of water deficiency on plant growth and physiology and the quality of fruits. The aim of this study is to explore the physiological mechanisms by which water deficiency affects tomato fruit quality. The findings are significant for understanding how water management can enhance the quality of tomato fruits.

## 2. Materials and Methods

### 2.1. Experimental Material

Tomato seeds (*Solanum lycopersicum* cv. Micro Tom) were soaked in deionized water and agitated in a Rotary Oscillator (HY-5A; Jintan Hengfeng Instrument Factory, Changzhou, China) for 24 h to achieve 90% pre-germination, allowing the radicle to emerge from the seed coat. The seeds were then surface-sterilized using 4% (*v*/*v*) sodium hypochlorite for 15 min and rinsed several times with sterile distilled water. Next, the seeds were sown in vermiculite and placed in a thermostat to germinate in the dark at 28 °C. After germination, the seedlings were transferred to an artificial climatic chamber (RDN-1000D-4; Ningbo Southeast Instrument Co., Ningbo, China) with a photoperiod of 14 h light and 10 h dark, a temperature regime of 28 °C during the day and 18 °C at night, 70% relative humidity, and a photosynthetic photon flux density of 300 µmol m^−2^ s^−1^. When the seedlings developed three fully expanded true leaves (after 8 days), healthy and uniformly growing plants were selected and transplanted into pots (10 × 10 × 10 cm), with one plant per pot. The seedlings were then maintained in the climatic chamber under the same conditions (14 h/10 h, 28 °C/18 °C). The substrate ratio was V commercial seedling substrate:V vermiculite:V perlite = 3:2:1.

### 2.2. Experimental Design

Tomato plants were artificial pollinated with a miniature vibrating pollinator. Fruits from the same flowering stage were marked. Irrigation treatments were applied at different frequencies during fruit expansion (20 d of pollination) until fruit maturity (45 d of pollination). The irrigation treatment frequencies were set as (1) CK: normal irrigation, irrigated once in 3 days; (2) T1: irrigated once in 4 days; (3) T2: irrigated once in 5 days; (4) T3: irrigated once in 6 days; (5) T4: irrigated once in 7 days; (6) T5: irrigated once in 8 days. CK was fully irrigated with a consistent substrate moisture content of 90% of the maximum FMC, and T1–T5 were maintained with a substrate moisture content of 80%, 70%, 60%, 50%, and 40% of the maximum FMC, respectively. The moisture content of the substrate was measured using the gravimetric method. Each treatment was replicated three times, with 15 plants per replicate. Watering took place at nine o’clock every morning, and the irrigation volume remained consistent across all treatments, with each pot receiving 150 mL of water during each irrigation event. The treatments were conducted until fruit ripening, and samples were taken for the determination of related indexes. Uniformly sized tomato fruits (30 fruits per treatment) were collected, frozen in liquid nitrogen, and subsequently stored at −70 °C for the purpose of determining the relevant indicators. The water deficit treatment with less stress damage to plants and the most significant effect on fruit quality improvement was selected based on plant growth indices, photosynthetic characteristics, chlorophyll fluorescence parameters, and fruit quality indices.

The screened treatment (T3) and the control CK were continued until the next batch of trials (August–December 2023), with the treatments set as (1) CK: normal irrigation, once in 3 d, and (2) MD: moderate water deficit: once in 6 d. CK was fully irrigated with a substrate moisture content of 90% of the maximum FMC, and MD with a substrate moisture content of 60% of the maximum FMC. The quality indexes of their treatments were determined at the mature green, the turning, and the ripening stages. Experimental materials, site, and sampling methods were as above.

### 2.3. Plant Growth Indexes

During the treatment processing, fifteen plants were randomly chosen from each treatment, and the plant height, stem thickness, and number of leaves of the tomato plants were tracked and recorded at 10, 20, and 30 D. Three plants from each treatment were selected to measure their total leaf area.

### 2.4. Gas Exchange and Chlorophyll Fluorescence Parameters of Tomato Leaves

The photosynthetic characteristics of the fifth true leaf of the tomato plants were measured through a portable photosynthetic system (CIRAS-2, PP System, Hitchin, UK) with light intensity of 1800 lx.

The chlorophyll fluorescence parameters were determined through a modulated chlorophyll fluorescence imaging system (MAXI Imaging-PAM, Walz, Germany). Before determination, the plants were placed under dark conditions for 30 min of dark adaptation. The first functional leaf of the plant was selected for assay, and three plants were measured for each treatment. The fluorescence parameters F0 (initial fluorescence under dark adaptation) and Fm (maximum fluorescence under dark adaptation) were detected with saturated pulsed light (2700 μmol m^−2^ s^−1^). Subsequently, the instrument was adjusted to a photochemical light intensity of 81 μmol m^−2^ s^−1^, light-adapted for 5 min, and a photochemical time of 0.8 s. The instrument was turned on every 20 s to determine F0′ (initial fluorescence in the condition of light adaptation), Fs (steady-state fluorescence in the condition of light adaptation), and Fm’ (maximum fluorescence under light adaptation). Y(II) = (Fm′ − Fs)/Fm′; Fv/Fm = (Fm − F0)/Fm; qP = (Fm′ − Fs)/(Fm′ − F0′); NPQ = (Fm − Fm′)/ Fm′.

### 2.5. Morphological Indicators, Dry Weight, and Water Content of Tomato Fruit

The measurement of the equatorial and longitudinal diameters of fruits was performed using vernier calipers. Single fruit weight was worked out by weighing three fruits from each treatment; fruit samples were killed at 105 °C for 30 min and then baked at 80 °C until they reached a constant weight to record the dry weight of the single fruits. Fruit water content (%) = [(fresh weight per fruit − dry weight per fruit)/fresh weight per fruit] × 100%. Tomato peel color parameters were measured by using a CR-10 Plus colorimeter (Konica Minolta, Inc., Tokyo, Japan).

### 2.6. Soluble Solids Content of Tomato Fruit

The soluble solids content of the fruit was measured using a hand-held refractometer (Atago Co. Ltd., Tokyo, Japan). The sample was cut at the equator and gently pressed to release seedless sample juice onto the center of the prism of a hand-held refractometer. If any seeds were present, they were discarded and this step was repeated. The sample was allowed to stand for 1 min to ensure it was evenly spread and free of air bubbles, then the reading was taken and recorded as a percentage.

### 2.7. Soluble Protein Content of Tomato Fruit

Coomassie Blue Staining Destaining Solution G-250 was used to measure the content of soluble protein [22]. A 0.5 g amount of fresh tomato samples was taken, 5 mL of ultrapure water was added, and the mixture was ground until homogenized. Then, it was poured it into a 10 mL centrifuge tube and stirred at 10,000 r·min^−1^ for 10 min. A 1 mL amount of supernatant was sucked up, and 5 mL of Coomassie Blue Staining Destaining Solution G-250 was added, mixed well, and left for two minutes. Then, the color at 595 nm was compared, the absorbance value was recorded, and the content of soluble proteins according to the standard curve was calculated. The standard substance used for this standard curve is Bovine Serum Albumin (BSA), measured in mg/mL. The results determined using the Coomassie Blue Staining Destaining Solution G-250 method are typically expressed as BSA equivalents.

### 2.8. Vitamin C Content of Tomato Fruit

Vitamin C content was titrated with 2,6-dichlorophenol indophenol sodium solution [23]. A 0.5 g amount of fresh tomato sample was accurately weighed, 1.5 mL of 2% oxalic acid was added, and the mixture was ground into a homogeneous slurry. Then, a small amount of 1% oxalic acid was added and the mixture was transferred to a 50 mL volumetric flask. A 0.5 mL amount of 30% zinc sulfate was added to the solution, which was then shaken well. Then, 0.5 mL of 15% potassium ferricyanide was added. It was diluted to 50 mL with 1% oxalic acid and filtered, and the filtrate was the solution for analysis. For the sample determination, 4 mL of the extract was taken and 2 mL of 2,6-dichlorophenolindophenol solution and 5 mL of xylene were added. It was shaken well and allowed to stand to separate into layers. The upper-layer solution was taken and its absorbance was measured at 500 nm using a UV-1800 UV spectrophotometer (SHIMADZU, Kyoto, Japan), calibrating to zero with xylene. Each treatment was repeated three times to obtain an average value.

### 2.9. Carotenoids Contents of Tomato Fruit

Carotenoid Fraction Analysis: This includes the determination of lycopene, α-carotene, β-carotene, and lutein. The carotenoid contents were assessed using a modified method from Zeb et al. [24]. A total of 0.5 g of lyophilized tomato powder was placed in a brown glass vial, to which 30 mL of a petroleum ether and acetone mixture (2:1, *v*/*v*) was added. The mixture was subjected to ultrasonic extraction at 30 °C for 40 min, or until the color faded completely. The extract was then transferred to a separatory funnel and washed twice with 250 mL of ultrapure water, and any residual aqueous phase was removed using anhydrous Na_2_SO_4_. The resulting extract was concentrated to dryness at 40 °C using a rotary evaporator and subsequently reconstituted in a mixture of 25 mL acetonitrile, dichloromethane, and methanol (55:20:25, *v*/*v*/*v*). The filtrate was passed through a 0.22 µm organic filter membrane prior to analysis via high-performance liquid chromatography (HPLC). The chromatographic parameters were as follows: The mobile phase consisted of methanol, acetonitrile, and dichloromethane in a ratio of 25:55:20 (*v*/*v*/*v*). An HPLC C18 column (250 mm × 4.6 mm, 5 µm, Waters Symmetry, Milford, MA, USA) was utilized, with the column temperature maintained at 30 °C and a flow rate of 1.2 mL/min. Qualitative analysis of the compounds was performed based on the retention times of various standard compounds (all carotenoid standards were purchased from Shanghai Yuanye Biotechnology Co., Ltd., Shanghai, China). The detection of each compound was conducted using a 3D channel in the HPLC instrument, with the following wavelengths used for detection: 450 nm for β-carotene and lutein, 470 nm for lycopene, and 286 nm for α-carotene. Quantitative calculations were made based on the standard curves. Data analysis was carried out using Empower software (version 3.7.0; Waters, Milford, MA, USA).

### 2.10. Sugar Compositions of Tomato Fruit

The extraction of sugar components was based on the method of Liu Fanhong [25], with slight modifications. A 5 g amount of tomato sample was weighed accurately, homogenized, transferred to a 50 mL centrifuge tube, and diluted to 25 mL with ultrapure water. The homogenate was ultrasonicated at 30 °C for 60 min, then centrifuged at 4 °C for 10,000× *g* for 10 min and filtered. A 2 mL amount of filtrate was extracted and filtered with 0.45 um microporous aqueous phase filter membrane. The filtrate was determined by high-performance liquid chromatography (HPLC). The analytes were detected by the Agilent Differential Occlusion Detector (Aglient series 1100, Santa Clara, CA, USA), and the chromatographic conditions were as follows: the mobile phase was 75% acetonitrile at a flow rate of 1.0 mL min^−1^, and the chromatographic column was LC-NH2 (4.6 mm × 250 mm) at 30 °C, with an injection volume of 25 μL. Three replicates were carried out in each treatment, and the average value was obtained. Qualitative analysis of the compounds was performed based on the retention times of various standard compounds, including glucose, fructose, and sucrose (all standards were purchased from Shanghai Yuanye Biotechnology Co., Ltd., Shanghai, China). The area of the peaks from the standard compounds was used to convert the area of the sample chromatographic peaks into the concentration or content of the sugars, expressed in mg/g.

### 2.11. Organic Acid Compositions of Tomato Fruit

The analysis of organic acid components was conducted following a modified method by Coelhoa et al. [26]. Fresh tomato samples weighing 5 g were homogenized and then transferred to a 50 mL centrifuge tube, diluted to 25 mL with ultrapure water, and thoroughly mixed. The mixture was centrifuged at 10,000 rpm for 10 min at 4 °C. After centrifugation, 2 mL of the supernatant was collected and filtered through a 0.22 µm membrane for high-performance liquid chromatography (HPLC) analysis. The analysis was performed using a high-performance liquid chromatograph equipped with a UV detector (Agilent 1260 Infinity II, Agilent Technologies, Santa Clara, CA, USA). All separations were conducted on an X-Peonyx AQ-C18 column (250 × 4.6 mm, Feini Gen Instrument, Guangzhou, China). The detection wavelength was set at 210 nm, with a mobile phase of 0.85% phosphoric acid, a flow rate of 1.2 mL/min, and a column temperature of 30 °C, with an injection volume of 5 µL. Qualitative analysis of the compounds was performed based on the retention times of various standard compounds, including α-ketoglutaric acid, malic acid, oxalic acid, ascorbic acid, tartaric acid, citric acid, and succinic acid (all standards were purchased from Shanghai Yuanye Biotechnology Co., Ltd., Shanghai, China). The area of the peaks from the standard compounds was used to convert the area of the sample chromatographic peaks into the concentration or content of the acids, expressed in mg/g.

### 2.12. Volatile Compounds of Tomato Fruit

The volatile components of tomato were analyzed by electronic nose (PEN3, Airsense Analytics GmbH, Schwerin, Germany) according to the method of Wang et al. [27] with minor modifications. A 5 g amount of fresh tomato was broken until homogenized and added to a headspace bottle with 1.5 g of anhydrous sodium sulphate which was capped tightly and then placed on a 70 °C magnetic stirrer and heated and stirred for 15 min to equilibrate the internal headspace gas. A syringe needle was then inserted into the headspace vial to measure volatile compounds. Electronic nose detection conditions were as follows: rinse time of 60 s, sensor zero time of 5 s, pre-sampling time of 5 s, injection flow rate of 400 mL·min^−1^, and a measurement time of 180 s. The measurement was carried out at the same time. The response characteristics and thresholds of the electronic nose sensor array are shown in Table 1 [28].

### 2.13. Statistical Analysis of Tomato Fruit

The results are presented as the mean ± standard error (SE). Data were analyzed using one-way ANOVA with SPSS 22.0 software (SPSS Institute Inc., Chicago, IL, USA), and significant differences were compared using Duncan’s multiple range test (*p* < 0.05). Origin 2021 (Origin Lab Institute Inc., Northampton, MA, USA) was used for mapping.

## 3. Results

### 3.1. Effect of Water Deficit on Growth Indices of Tomato

With the reduction in irrigation frequency, tomato plant height, stem thickness, leaf area, and number of leaves in the same period showed a downward trend, and were all highest in the control CK. The lower the frequency of irrigation, the more significant the difference (Table 2). In the whole growth period, the plant height, stem diameter, leaf area, and leaf number of the T4 and T5 treatments were decreased compared with the control, and the difference was significant. The difference was the most significant in the T5 treatment, where the indices were reduced by 12.21%, 10%, 43.67%, and 14.58% compared with the control CK at 20 days of treatment, respectively.

### 3.2. Effect of Water Deficit on Photosynthetic Indexes of Tomato Leaves

**Figure 1:**
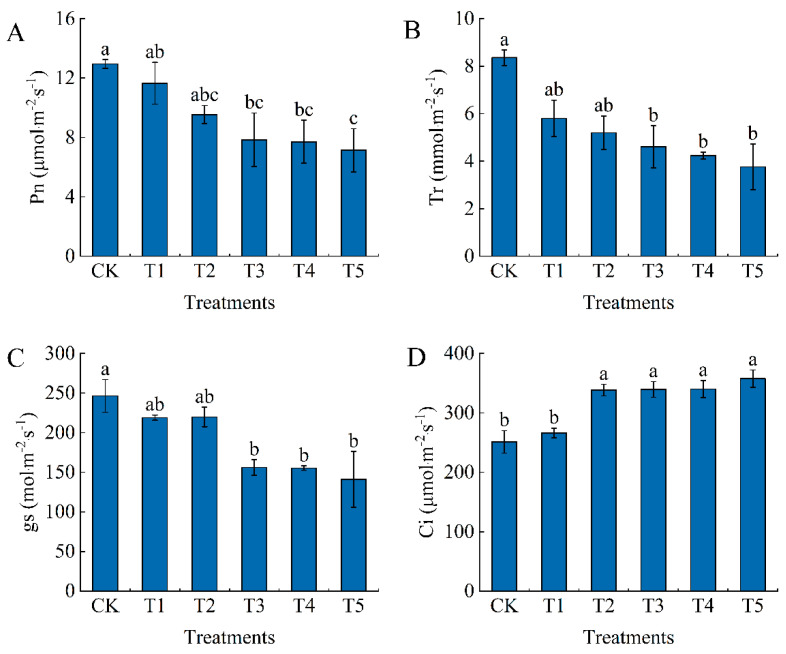
Effects of water deficit on photosynthetic characteristics of tomato leaves. (**A**) Net photosynthetic rate (Pn). (**B**) Transpiration rate (Tr). (**C**) Stomatal conductance (gs). (**D**) Intercellular carbon dioxide concentration (Ci). CK: normal irrigation, spontaneously once in 3 days; T1: irrigating once in 4 days; T2: irrigating once in 5 days; T3: irrigating once in 6 days; T4: irrigating once in 7 days; T5: irrigating once in 8 days. The histogram features a short vertical line denoting the mean ± standard error (*n* = 3), with a significance threshold of *p* < 0.05. Distinct lowercase letters indicate the variation in treatment effects.

The net photosynthetic rate (Pn), transpiration rate (Tr), and stomatal conductance (gs) of the control treatment were higher than those of the other treatments, and the differences were greater with the greater extent of water deficit (Figure 1A–C). The T1 and T2 treatments were reduced compared with CK, but the differences were not significant. Among them, the T5 treatment had the lower Pn, Tr, and gs values, which were reduced by 44.86%, 55.02%, and 42.81%, respectively, compared with CK. The intercellular CO_2_ concentration (Ci) of the control was the minimum, and it displayed an increasing trend as the water deficit increased. The T2, T3, T4, and T5 treatments were significantly different from the control CK, with 34.67%, 35.16%, 35.39%, and 42.41% higher Ci than CK, respectively (Figure 1D).

### 3.3. Effect of Water Deficit on Chlorophyll Fluorescence Parameters of Tomato Leaves

**Figure 2:**
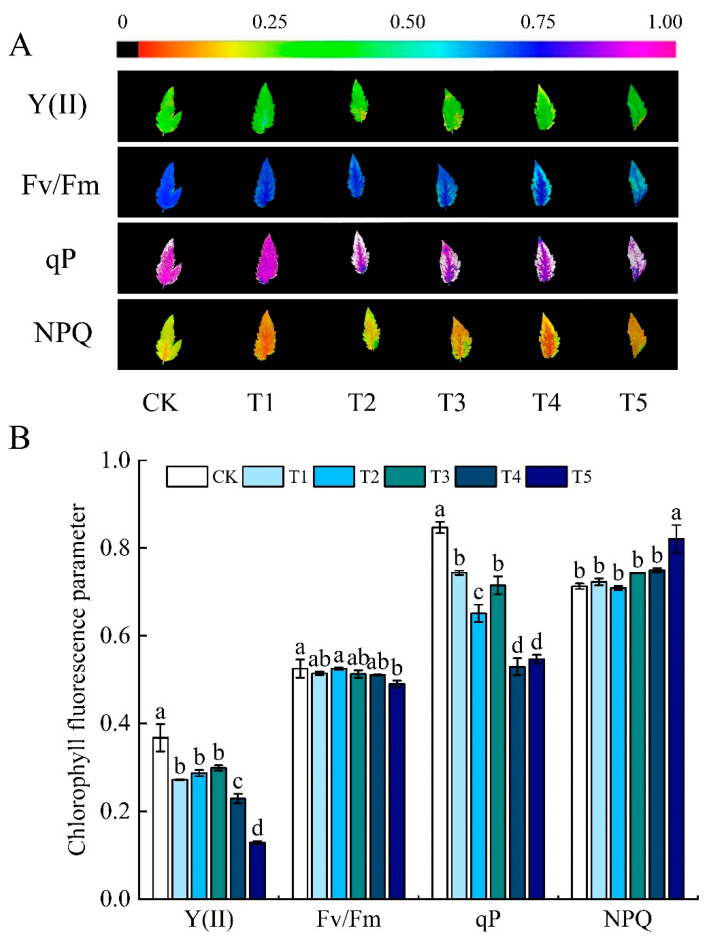
Effects of different water deficits on chlorophyll fluorescence parameters of tomato leaves. (**A**) Chlorophyll fluorescence imaging; (**B**) chlorophyll fluorescence parameters; Y (II): actual photochemical efficiency; NPQ: non-photochemical burst factor; Fv/Fm: maximum photochemical efficiency; qP: photochemical burst factor. CK: normal irrigation, spontaneously once in 3 days; T1: irrigating once in 4 days; T2: irrigating once in 5 days; T3: irrigating once in 6 days; T4: irrigating once in 7 days; T5: irrigating once in 8 days. The histogram features a short vertical line denoting the mean ± standard error (*n* = 3), with a significance threshold of *p* < 0.05. Distinct lowercase letters indicate the variation in treatment effects.

The actual photosynthetic efficiency of PSII (Y (II)), the maximum photochemical efficiency (Fv/Fm), and the photochemical quenching coefficient (qP) in tomato leaves showed decreasing trends under water deficit (Figure 2B). The Y (II) in all treatments decreased by 27.03%, 21.62%, 18.92%, 37.84%, and 64.86% by contrast with that of the control, respectively. The Fv/Fm of the T5 treatment was the minimum, which significantly decreased by 7.55% compared with the control. The qP of the T4 treatment was the minimum, which was significantly reduced by 37.65% compared with CK. The non-photochemical quenching coefficient (NPQ) showed an increasing trend. The NPQ value was maximum in the T5 treatment, and was significantly higher compared with the control.

### 3.4. Effect of Water Deficit on the Appearance and Morphology of Tomato Fruits

**Figure 3:**
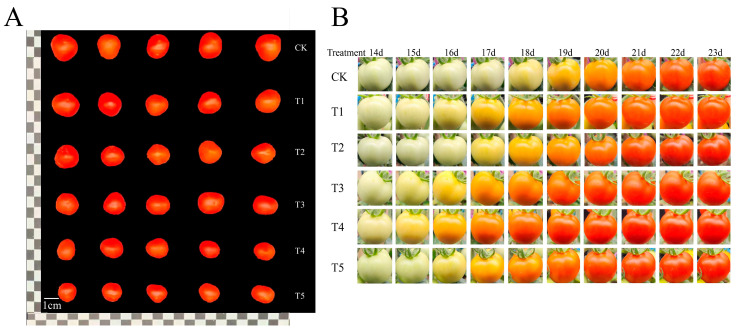
Effect of water deficits on the appearance of tomato fruits. (**A**) Photograph of the appearance and morphology of tomato fruits during the mature stage; (**B**) The changes in the appearance and morphology of tomato fruits over different days of treatment. CK: normal irrigation, spontaneously once in 3 days; T1: irrigating once in 4 days; T2: irrigating once in 5 days; T3: irrigating once in 6 days; T4: irrigating once in 7 days; T5: irrigating once in 8 days.

The transverse and longitudinal diameter and dry and fresh weight of a single fruit treated with CK were at their maximum, with no significant differences between the T1, T2, and T3 treatments (Table 3). The transverse diameter, longitudinal diameter, single fruit fresh weight, single fruit dry weight, and fruit water content of T4 and T5 were significantly different from those of CK, being reduced by 15.28%, 4.23%, 39.48%, 22.95%, and 4.60%, respectively, in T4 and by 17.76%, 12.13%, 46.14%, 29.51%, and 5.75%, respectively, in T5 compared with CK. Different levels of water deficit treatment have varying effects on the size and shape of tomatoes. It was observed that mild and moderate water deficits had minimal impact on the size of the fruits, while severe water deficit treatments significantly reduced the size of the tomatoes, affecting their weight (Figure 3A). Additionally, it was found that water deficit treatments can promote earlier color development and maturation of the fruits (Figure 3B).

### 3.5. Effect of Water Deficit on Color Parameters of Tomato Fruits

**Figure 4:**
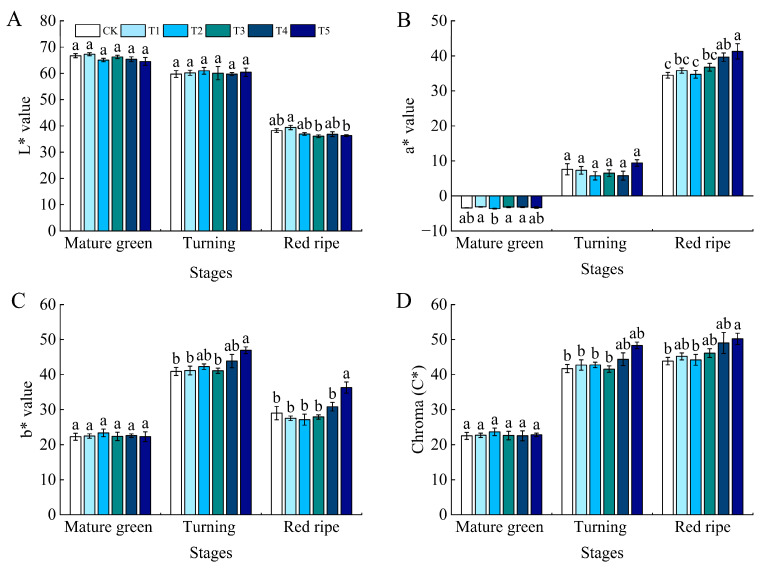
Effect of water deficit on the color of tomato fruit skin. (**A**) Brightness parameter (L*); (**B**) red and green parameters (a*); (**C**) yellow and blue parameters (b*); (**D**) color saturation (C*). CK: normal irrigation, spontaneously once in 3 days; T1: irrigating once in 4 days; T2: irrigating once in 5 days; T3: irrigating once in 6 days; T4: irrigating once in 7 days; T5: irrigating once in 8 days. The histogram features a short vertical line denoting the mean ± SE (*n* = 3), with a significance threshold of *p* < 0.05. Distinct lowercase letters indicate the variation in treatment effects.

There was no significant difference in the brightness parameters (L*) of tomato fruits in the mature green and the turning stages. In the ripening stage, the L* value of tomato fruits under the T1 treatment was the maximum, and was significantly different from those in the T3 and T5 treatments. As the tomato fruits ripened, the brightness parameters tended to decrease (Figure 4A). The red–green parameter (a*) was the minimum in the mature green stage with treatment T2, and the difference was significant with all other treatments. At the turning stage, treatment T5 was the maximum, but there was no significant difference between the treatments. At the ripening stage, the control was the minimum, and treatment T5 was the maximum. There was no significant difference between the control and the treatments T1, T2, and T3, and significant differences with treatments T4 and T5. The difference in a* value tended to grow significantly as the fruits ripened (Figure 4B). The yellow–blue color (b*) difference of each treatment was not significantly different at the mature green stage, and, at the turning stage, the control was the minimum and the T5 treatment was the maximum. The difference between the control and the T5 treatment was significant, and the difference with the other treatments was not significant. At the ripening stage, the value of the T5 treatment was the maximum, and there was a significant difference with all treatments, which was improved by 25.19% compared with the control (Figure 4C). The color light value parameter (C*) showed an increasing trend with fruit ripening, but there was no significant difference between treatments at the mature green and turning stages. At the ripening stage, the T5 treatment had the maximum value, which was significantly different from the control and T2 treatments, and increased by 14.56% compared to the control (Figure 4D).

### 3.6. Effect of Water Deficit on the Nutritional Quality of Tomato Fruits

**Figure 5:**
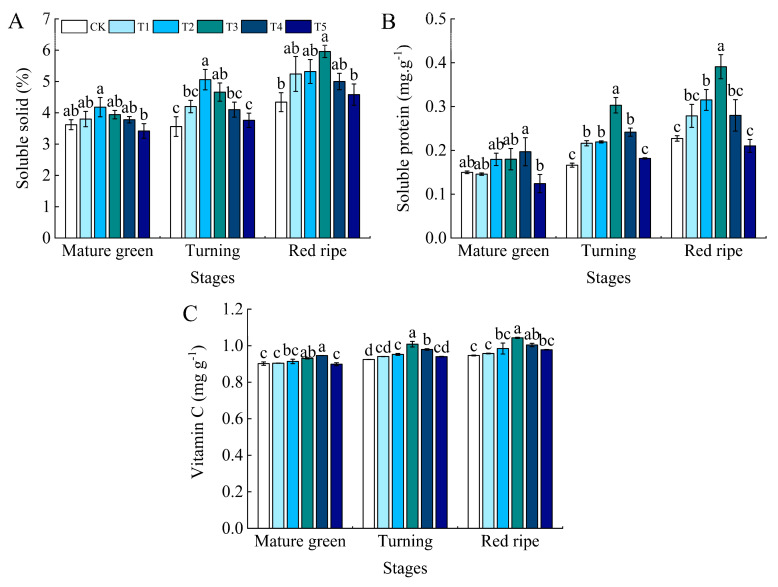
Effect of water deficit on soluble solid, soluble protein, and vitamin C contents in tomato fruits. (**A**) Soluble solid content; (**B**) soluble protein content; (**C**) vitamin C content. CK: normal irrigation, spontaneously once in 3 days; T1: irrigating once in 4 days; T2: irrigating once in 5 days; T3: irrigating once in 6 days; T4: irrigating once in 7 days; T5: irrigating once in 8 days. The histogram features a short vertical line denoting the mean ± SE (*n* = 3), with a significance threshold of *p* < 0.05. Distinct lowercase letters indicate the variation in treatment effects.

As the tomato fruits matured, the soluble solids, soluble proteins, and vitamin C content in all treatment groups showed an upward trend (Figure 5).

During the mature green stage, treatment T2 had the highest soluble solids content (Figure 5A), while treatment T5 had the lowest. There was a significant difference between T2 and T5, with T2 increasing by 15.47% and 22.22% compared to the control and T5, respectively. In the turning stage, T2 also exhibited the highest levels, while CK had the lowest, with T2 significantly increasing by 42.13% compared to CK. In the ripening stage, treatment T3 had the highest soluble solids content, showing a 37.33% increase compared to the control.

Soluble protein content (Figure 5B) was at its maximum in the T4 treatment at the mature green stage, which was significantly different from the T5 treatment but not significantly different from the other treatments, and increased by 33.33% compared to the control. It was maximum in the T3 treatment at both turning and ripening stages, and the difference was significant between the T3 treatment and each of the treatments, being significantly increased by 76.47% and 69.57% compared to the control, respectively.

Vitamin C content (Figure 5C) was maximum in the T4 treatment at the mature green stage, with a non-significant difference compared with the T3 treatment, but a significant difference compared with all other treatments. The T4 treatment increased by 5.55%, 2.15%, and 5.56% compared to the CK, T3, and T5 treatments, respectively. The T3 treatment was the maximum at both turning and ripening stages and differed significantly from all other treatments, with a significant increase of 9.78% and 9.47% over the control, respectively.

Based on the analysis of the above experiment results, we screened out the water deficit treatments that had less damage due to plant stress and the most significant effect on fruit quality in terms of plant growth indexes, photosynthetic characteristics, chlorophyll fluorescence parameters, and fruit quality indexes and further investigated the effects of these treatments on the content of carotenoids, sugar components, organic acid components, and volatile compounds in the ripening process of tomato fruits.

### 3.7. Effect of Moderate Water Deficit on the Bioactive Compounds in Tomato Fruits

**Figure 6:**
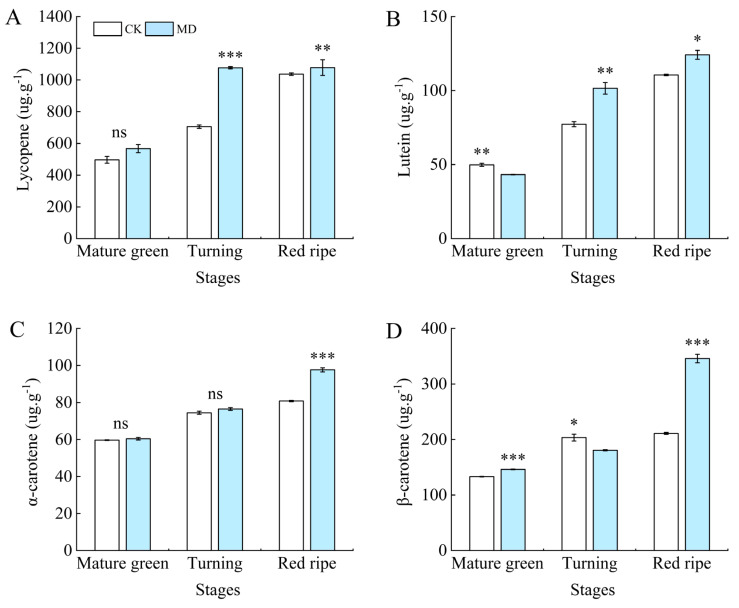
Effect of moderate water deficit on carotenoids in tomato fruits. (**A**) Lycopene; (**B**) lutein; (**C**) α-carotene; (**D**) β-carotene. CK: normal irrigation, spontaneously once in 3 days; MD: irrigating once in 6 days. The histogram includes a short vertical line that indicates the mean ± SE (*n* = 3), while asterisks highlight significant differences between treatments based on the Student’s *t*-test (* *p* < 0.05, ** *p* < 0.01, *** *p* < 0.001, ns: There was no significant difference).

The contents of lycopene, lutein, α-carotene, and β-carotene in tomato fruits showed an increasing trend with the development and maturity of the fruits (Figure 6). At the mature green stage, the lycopene content of the MD treatment was not significantly different from that of the control. At the turning and ripening stages, it was significantly different from the control, being significantly increased by 52.56% and 11.85% compared with the control CK (Figure 6A). Lutein content was higher in CK and significantly different from MD at the mature green stage. During the turning and ripening stages, the lutein content of the MD treatment was higher, being significantly increased by 31.42% and 12.28% compared with CK (Figure 6B). The α-carotene content was higher in treatment MD during the mature green and turning stages, but it did not differ significantly from the control (CK). However, at the ripening stage, treatment MD showed a significant increase of 20.87% compared to the control (CK) (Figure 6C). At the mature green stage, β-carotene content was higher under treatment MD, being significantly higher by 9.74% compared to CK. At the turning stage, it was higher under the control CK, being significantly higher by 12.65% compared to treatment MD. At the ripening stage, the content was significantly higher by 63.89% under the MD treatment compared to the control (Figure 6D).

### 3.8. Effect of Moderate Water Deficit on the Content of Sugar Components in Tomato Fruits

**Figure 7:**
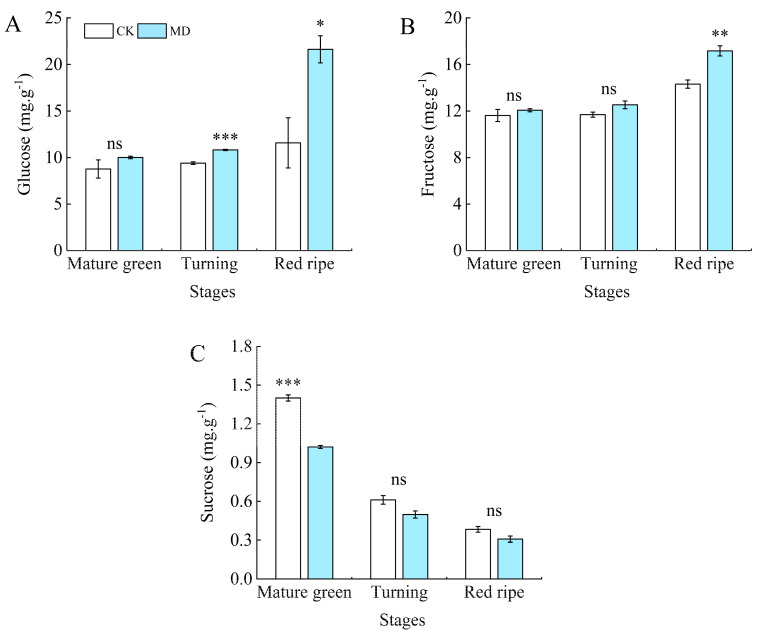
Effect moderate water deficit on the content of sugar components in tomato fruits. (**A**) Glucose; (**B**) fructose; (**C**) sucrose. CK: normal irrigation, spontaneously once in 3 days; MD: irrigating once in 6 days. The histogram includes a short vertical line that indicates the mean ± SE (*n* = 3), while asterisks highlight significant differences between treatments based on the Student’s *t*-test (* *p* < 0.05, ** *p* < 0.01, *** *p* < 0.001, ns: There was no significant difference).

Fructose and glucose contents were higher in the later stages of tomato development and sucrose was high in the early stages of growth (Figure 7). Fructose and glucose contents reached their maximum values at the ripening stage, and sucrose content reached its maximum level at the mature green stage. The fructose content of tomatoes at the same developmental stage showed an increasing trend with water deficit, with no significant difference between the control and MD treatments at the mature green stage and significant differences at the turning and ripening stages, with MD treatments being increased by 7.18% and 19.83%, respectively, compared with the control (Figure 7B). The glucose content increased significantly by 14.14%, 15.11%, and 86.70% in comparison to the control at the mature green, turning, and ripening stages, respectively (Figure 7A). The sucrose content of the tomatoes gradually decreased with fruit ripening. Compared with the control, MD treatment decreased by 27.14%, 18.03%, and 18.42%, respectively (Figure 7C).

### 3.9. Effect of Moderate Water Deficit on the Content of Organic Acid Components in Tomato Fruits

**Figure 8:**
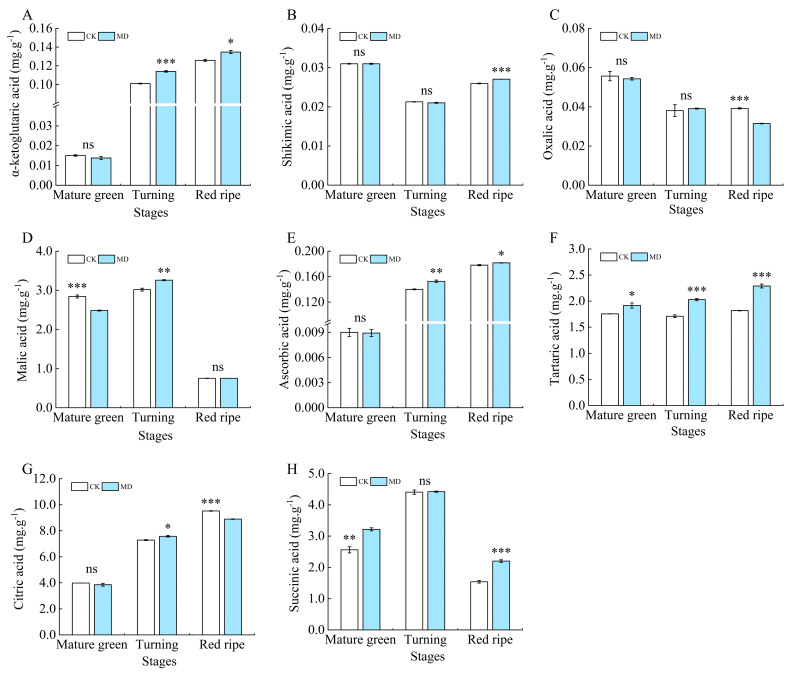
Effect of moderate water deficit on the content of organic acid fractions in tomato fruits. (**A**) α-ketoglutaric acid; (**B**) shikimic acid; (**C**) oxalic acid; (**D**) malic acid; (**E**) ascorbic acid; (**F**) tartaric acid; (**G**) citric acid; (**H**) succinic acid. CK: normal irrigation, spontaneously once in 3 days; MD: irrigating once in 6 days. The histogram includes a short vertical line that indicates the mean ± SE (*n* = 3), while asterisks highlight significant differences between treatments based on the Student’s *t*-test (* *p* < 0.05, ** *p* < 0.01, *** *p* < 0.001, ns: There was no significant difference).

During the development of the tomato fruits, the contents of malic acid and succinic acid increased first and then decreased (Figure 8D,H). The contents of oxalic acid showed a decreasing trend in general (Figure 8C), and the contents of α-ketoglutaric acid, ascorbic acid, tartaric acid, and citric acid showed an increasing trend (Figure 8A,E–G). Shikimic acid content decreased first and then increased (Figure 8B). The content of citric acid in the fruit decreased significantly by 3.27% and 6.51% in treatment MD compared with the control at the mature green and ripening stages (Figure 8G). The content of malic acid in fruits decreased to the lowest at the maturity stage, and there was no significant difference between the MD treatment and the control (Figure 8D). There was no significant difference in oxalic acid content between the MD treatment and the control at the mature and turning stages, and the MD treatment significantly decreased by 17.95% compared with the control at the ripening stage (Figure 8C). The tartaric acid content in the MD treatment was notably greater than that in the control during the mature green, turning, and ripening stages, with increases of 9.09%, 18.71%, and 25.82%, respectively (Figure 8F). The content of succinic acid showed an overall trend of increasing and then decreasing with the development of the fruit. The content of succinic acid was the lowest in each treatment at ripening stages, and the difference among treatments was significant (Figure 8H). There was no significant difference in shikimic acid between treatments at the mature green and turning stages. At the ripening stage, the MD treatment was significantly increased by 3.85% compared with the control (Figure 8B). The content of α-ketoglutaric acid in MD was significantly increased by 10% and 8.33% compared with the control at the turning and ripening stages (Figure 8A). The content of ascorbic acid was higher in the whole development period, and the content of the MD treatment was significantly increased by 7.14% and 5.88% compared with the control at the turning and ripening stages (Figure 8E). Moderate water deficit improved the fitting degree of acid components to varying degrees.

### 3.10. Effects of Moderate Water Deficit on Total Soluble Sugar, Organic Acid Content, and Sugar–Acid Ratio of Tomato Fruit

**Figure 9:**
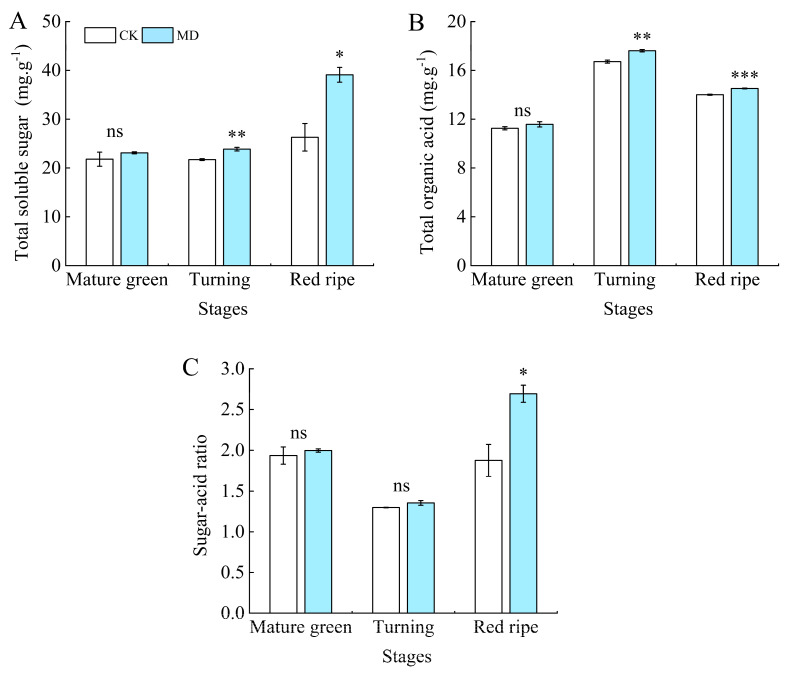
Effect of moderate water deficit on the content of total soluble sugar, organic acid, and sugar–acid ratio in tomato fruits. (**A**) Total soluble sugar; (**B**) total organic acid; (**C**) sugar–acid ratio. CK: normal irrigation, spontaneously once in 3 days; MD: irrigating once in 6 days. The histogram includes a short vertical line that indicates the mean ± SE (*n* = 3), while asterisks highlight significant differences between treatments based on the Student’s *t*-test (* *p* < 0.05, ** *p* < 0.01, *** *p* < 0.001, ns: There was no significant difference).

The total soluble sugar content rose as the fruit ripened. At every growth stage, the MD treatment showed higher levels compared to the control, with significant differences observed during the turning and ripening stages. Specifically, the MD treatment increased by 9.90% and 48.69% compared to the control at these stages, respectively (Figure 9A). In the whole fruit development process, the total organic acid content demonstrated a pattern of first rising and then falling. At the turning and ripening stages, the MD treatment was significantly different from the control, being increased by 5.32% and 3.64%, respectively (Figure 9B). The ratio of sugar to acid exhibited an initial decrease followed by an increase throughout fruit development. There was no significant difference between treatments at the mature green and turning stages. The MD treatment at the ripening stage was significantly increased by 43.09% compared with the control (Figure 9C).

### 3.11. Effects of Moderate Water Deficit on Volatile Substances in Tomato Fruit

**Figure 10:**
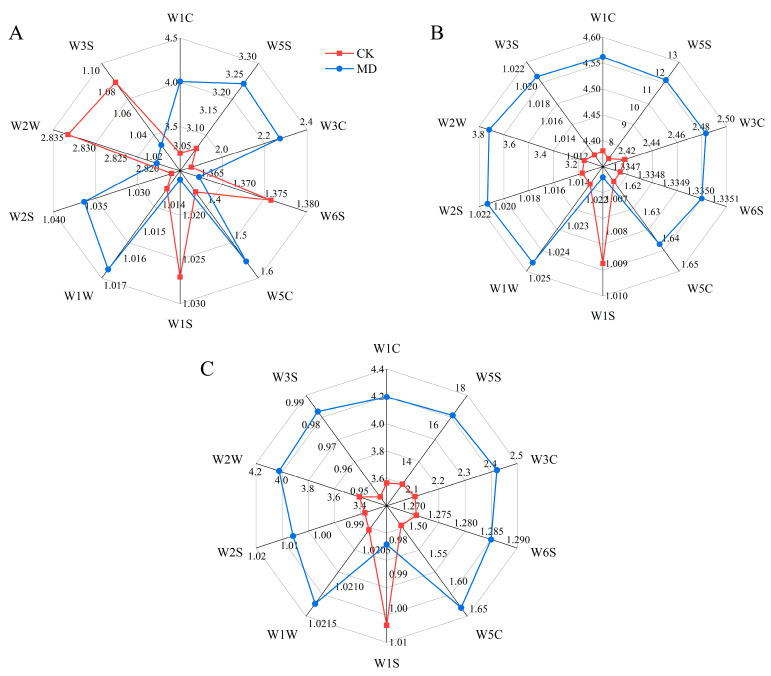
Effect of water deficit on volatile substances in tomato fruits. (**A**) Mature green stage; (**B**) turning stage; (**C**) ripening stage. CK: normal irrigation, spontaneously once in 3 days; MD: irrigating once in 6 days.

The difference of the characteristic response radar map of the sensor array reflects the difference of the aroma of the sample to a certain extent (Table 1). The radar map of the average response value of the electronic nose in the course of tomato fruit maturation is shown (Figure 10). At the mature green stage, the fruit had the strongest response to the W1C and W5S sensors. Under moderate water deficit treatment, the response values of the W1C, W5S, W3C, W5C, W1W, and W2S sensors were increased by 25%, 5.88%, 22.70%, 13.97%, 0.30%, and 0.97%, respectively, compared with the control, and the response values of the W6S, W1S, W2W, and W3S sensors were reduced by 0.73%, 0.98%, 0.35%, and 5.56%, respectively, compared with the control (Figure 10A).

At the turning stage, under the treatment of moderate water deficit, the response values of 10 sensors in the fruit were improved to varying degrees. The MD treatment significantly increased the response values of the fruit’s W5S and W2W sensors by 60.40% and 19.87%, respectively, compared to the control. There was no significant difference in the response value of the fruit on other sensors compared with the control at the significant level (Figure 10B).

At the ripening stage, the response value of the W5S sensor under moderate water deficit treatment was the highest, and was significantly increased by 28.40% compared with the control, while the response values of the W3C, W5C, W2S, and W2W sensors were significantly increased by 14.69%, 9.40%, 2.02%, and 17.89%, respectively, compared with the control. The response value of the W1S sensor was reduced by 2.97% under the MD treatment compared with the control (Figure 10C).

## 4. Discussion

In recent decades, climate change and severe drought have further aggravated the scarcity of fresh water resources in the world, resulting in severe water shortage in many areas [29]. At the same time, with the development of society, people’s demand for the nutritional quality of tomato fruits is also increasing. Therefore, the adoption of water-saving cultivation techniques has become the mainstream mode of tomato cultivation under the premise of rationally utilizing limited water resources, improving water utilization efficiency, and promoting tomato fruit quality [30,31]. Under water deficit conditions in plants, the root system will prioritize the allocation of more photosynthetic products for its own growth, thus reducing the supply of nutrients to above-ground parts such as leaves and stems, and the growth of above-ground parts will be relatively inhibited to a certain extent due to the reduced supply of photosynthetic products [32]. In this study, the plant height and stem diameter of all water deficit treatments were lower than that of the full irrigation CK treatment, and the inhibition became more serious with the increase in water deficit degree. Studies showed that severe water stress (up to 40% of the water capacity in the soil) significantly inhibited the plant height and stem thickness of eggplant compared to those of control [33]. Meanwhile, plant leaves are of vital importance for plant growth and development. As an important photosynthetic organ of plants, leaves provide essential nutrients and regulate the internal environment through photosynthesis and related physiological processes, thus promoting normal growth and development [34]. Plant response to environmental conditions, especially to water deficit, is the most limiting stress for growth and has impacts on the structure of tomato leaves. This study’s results demonstrated that water deficit inhibited the growth of tomato leaves, which led to a reduction in leaf area. Studies have shown that, after water deficit, the number and size of tomato leaves are reduced [35]. Other studies also showed that the leaf area of wheat treated with water deficit decreased [36].

Crops achieve survival under water scarcity conditions through the synergistic action of a range of physiological and morphological mechanisms. This is inseparable from the important role of water in photosynthesis, which requires water as a raw material, and plants rely on photosynthesis for nutrients and energy. At the same time, there is a certain interaction effect between photosynthesis and the chlorophyll fluorescence parameters of crops; changes in the water status of leaves affect both photosynthetic rate and fluorescence parameters [37]. A tomato plant closes its stomata under drought stress and reduces CO_2_ absorption, which will decrease the photosynthetic rate [38]. It was discovered that water deficit decreased stomatal conductance, transpiration rate, and maximum photochemical efficiency Fv/Fm and increased the volume of non-photochemical burst coefficient NPQ in peanut, confirming that incident energy is dispersed in the direction of heat dissipation rather than photochemistry under water deficit stress [39]. The experiment on tomato plants showed that the Pn and gs of tomato leaves decreased with the degree of water stress, and Ci increased. From the seedling stage to maturity, Fv/Fm and Y (II) showed a downward trend under each water stress treatment, and the more serious the water stress, the greater the decrease, while NPQ increased [40]. The results of this study also found that Y (II), qP, and Fv/Fm in the PSII systems and the Pn, Tr, and gs of tomato leaves showed a decreasing trend with water deficit, and the more serious the deficit, the more obvious the decline in these indexes. This indicated that the PSII reaction centers of tomato leaves were injured due to drought stress, their potential activity and light energy conversion efficiency were markedly weakened, and photoinhibition of photosynthesis emerged, while Ci and NPQ, on the other hand, showed an increasing trend with water deficit. It has been demonstrated that Y (II), Fv/Fm, and qP in the PSII systems and the Pn, E, and gs of rice leaves decreased with decreasing soil water content during the growing season, whereas the NPQ changed in the opposite manner [41].

Tomato fruit appearance indicators such as transverse diameter, longitudinal diameter, and single fruit weight are common indicators used to determine the growth status of the plant. Both the fruit growth rate and yield of tomato are affected by the fruit transverse diameter. A larger transverse diameter of the tomato reveals a faster growth rate, and the yield is relatively higher. The longitudinal diameter of the tomato is a determining factor affecting the morphology of the fruit, and the size of the longitudinal diameter also affects the weight of a single fruit [42]. The findings of this study revealed that there were no significant differences in fruit transverse or longitudinal diameters, or single fruit weights, in the mild water deficit treatments (T1, T2) or moderate water deficit treatment (T3) compared to the control, but they were significantly lower in the severe water deficit treatments (T4, T5). However, fruit water content decreased gradually with water deficit, especially under moderate and severe water deficit treatments, which were significantly decreased compared to control group. During apple fruit growth, heavy water stress caused inhibition of the weight and water content of the fruit at the final stage of fruit cell division, and apple fruit size development could also be inhibited by deficit irrigation [43,44].

In this present research, it was found that water deficit increased the a* value, b* value, and color saturation (C) of tomato fruits to varying degrees during ripening, with some reduction in peel brightness. The red colors of pomegranate (*Punica granatum* L.) pericarp increased under drought stress, while brightness was negatively correlated [45]. Water deficit had a beneficial influence on the color parameters of tomato fruit, with higher a* and b* values, less effect on L* values, and lower C in fruits [46,47]. The content and relative proportion of pigments such as chlorophyll and carotenoid in peel and pulp are the basis of fruit coloration [48]. In the process of fruit ripening, the color of tomato changed from green to red, and the color parameters of the peel also changed. The changes of internal substances were mainly manifested in the gradual accumulation of carotenoid components such as lycopene and β-carotene. Moderate water deficit accelerated this transformation process, and significantly accumulated carotenoid content, thus deepening the color of tomato fruit. Carotenoids are also indicators of tomato quality and have antioxidant effects. Carotenoid-rich fruits and vegetables are considered to be conducive to health and can decrease the risk of various diseases, especially certain cancers and eye diseases [49]. The most studied carotenoids are α-carotene, beta-carotene, lycopene, and lutein. An experiment showed that the carotenoid content of sunflower (*Helianthus annuus* L.) was higher under moderate and severe drought stress [50]. It was also observed that tomatoes treated with water deficit had significantly higher lycopene content [51].

Soluble protein and soluble solids contents in tomato fruits are vital indexes of nutrient quality, and soluble protein is the substance mainly used by plants for osmotic regulation [52]. Soluble solids play a crucial role in determining the flavor, texture, and moisture content of fruit. Consequently, increasing the soluble solids concentration in tomatoes can significantly improve their quality and market value [53]. In this present study, it was observed that soluble protein and soluble solids contents gradually increased with water deficit but decreased at severe water deficit levels. This may be due to water deficiency, which increases the osmotic pressure within the tomato fruit cells. To maintain osmotic balance inside the cells, the plants accumulate more solutes, such as soluble solids and soluble proteins [20]. Similarly, in an experiment on kiwifruit (*Actinidia deliciosa* (*A. Chev.*), it was found that water stress (soil water content 15–20%) increased the soluble solids and sucrose content of kiwifruit [54]. Vitamin C, as an antioxidant with the ability to scavenge free radicals and regenerate vitamin e, can perform an essential protective function in the body [55]. In the present study, the vitamin C content of the tomato fruits increased under water deficit. It is possible that, when tomato plants face stress from drought or other adverse conditions, their defense mechanisms are triggered, enhancing the synthesis of secondary metabolites, including vitamin C. This may result in an increase in vitamin C content. It was found that vitamin C content in amaranth increased significantly with increasing levels of drought stress [56]. It has also been shown that, in tomatoes, the concentration of vitamin C increased due to water limitation [30]. Interestingly, Massot et al. [57] proposed that the vitamin C content might initially decline during cell division and expansion due to the dilution effect but subsequently increase during ripening as hexose levels rise. The content of sugar components and organic acid components, which are major factors in tomato fruit flavor, is controlled by development and is reported to increase during ripening [58]. Water deficiency triggers various physiological responses, including reduced photosynthesis and disrupted nutrient transport, resulting in the accumulation of metabolites such as sugars and organic acids. In this study, it was found that the fructose and glucose contents of each treatment at the ripening stage were higher than those at the mature green and turning stages, while sucrose contents were the lowest at the ripening stage. Meanwhile, the fructose and glucose contents in the moderate water shortage treatment were significantly higher than those in the control treatment, while sucrose contents were lower than those in the control. This is consistent with the research results on the soluble sugars of grapes [59], strawberries [60], and different varieties of tomatoes [61] under mild and moderate water stress. At all stages, citric acid is the major organic acid, but unripe green tomatoes may contain high levels of malic acid. Conversely, malic acid levels in ripe fruit are quite low [62]. The present study also found that the organic acids in the tomato fruits consisted mainly of malic, tartaric, and citric acids, with citric acid content being the highest at all stages of fertility, and malic acid content decreasing during ripening. Except for oxalic and citric acid content, which decreased under water deficit at the ripening stage, all other organic acid contents increased under moderate water deficit, and total organic acid content also increased. Studies have shown that the organic acid content of tomatoes under different irrigation treatments is considerably higher than that of control tomatoes at a significant level [17]. However, the fact that the organic acid content of the tomato fruit has increased does not imply that the acidity of the tomato fruit taste is strengthened, as the absolute quantity and balance of sugars and acids jointly determine the taste of the fruit [63]. The electronic nose (E-nose) is an instrument engineered to replicate human olfactory perception, enabling the detection and classification of volatile mixtures without the olfactory fatigue often experienced by humans [64]. In our study, we used electronic nose technology to analyze the composition of volatile compounds in tomatoes under different water treatment conditions. The results indicated that the primary volatile compounds in tomatoes included nitrogen oxides (primarily detected by the W5S sensor), aromatic compounds (W1C sensor), aromatic organic sulfides (W2W sensor), aromatic amines (W3C sensor), and long-chain alkanes (W3S sensor). These compounds showed higher sensitivity in response to the electronic nose sensors. This is similar to the results obtained by Vanoli et al. [65]. Compared to the control, the moderate water deficit treatment led to an increase in the content of these volatile compounds in tomatoes, resulting in a more fragrant flavor profile for the fruit. Water deficit irrigation (with a field water content of 65%) also significantly enhanced the response values of the electronic nose sensors for the tomato samples [66]. Additionally, supplemental night lighting also increased the response of sensors such as W5S, W1C, and W3S in tomato fruits [67]. In summary, this study shows that, under water deficit conditions, the content of certain volatile compounds in tomatoes increases, and these components are precisely those detected by the electronic nose sensors. These changes ultimately alter the aromatic characteristics of tomatoes, demonstrating that water management can significantly impact tomato fruit flavor and providing a reference for further research on the effects of water management on tomato flavor.

## 5. Conclusions

The results of this study indicated that moderate water deficit treatments had less of an effect on tomato plant growth, while water deficit treatments were favorable for increasing the content of soluble solids, soluble proteins, and vitamin C in tomato fruits and promoting fruit quality. Moreover, on the basis of not affecting the fruit size and color, the moderate water deficit treatment (MD) significantly increased the content of carotenoid components, sugar components, and organic acid components in tomato fruit, improved the flavor quality of the fruit, and improved the aroma characteristics of the fruit; therefore, it was the optimal water deficit treatment. This study provides valuable insights into deficit irrigation methods for managing tomato quality to enhance consumer satisfaction. Future research should aim to investigate the molecular mechanisms through which moderate water deficits enhance the nutritional and flavor profiles of tomatoes.

## Figures and Tables

**Table 1:** The response features of the sensor array.

Number in Array	Sensor Name	Object Substances for Sensing	Threshold Value (mL·m^−3^)
R1	W1C	Aromatics	10
R2	W5S	Nitrogen oxides	1
R3	W3C	Ammonia and aromatic molecules	10
R4	W6S	Hydrogen	100
R5	W5C	Methane, propane, and aliphatic nonpolar molecules	1
R6	W1S	Broad methane	100
R7	W1W	Sulfur-containing organics	1
R8	W2S	Broad alcohols	100
R9	W2W	Aromatics, sulfur- and chlorine-containing organics	1
R10	W3S	Methane and aliphatics	10

**Table 2:** Effect of water deficit on tomato growth index.

Treatments	Plant Height (cm)			Stem Diameter (mm)			Leaf Area (cm^2^)			Number of Leaves		
	10 D	20 D	30 D	10 D	20 D	30 D	10 D	20 D	30 D	10 D	20 D	30 D
CK	9.83 ± 0.30 ^a^	10.06 ± 0.33 ^a^	10.28 ± 0.33 ^a^	5.15 ± 0.12 ^a^	5.3 ± 0.12 ^a^	5.64 ± 0.11 ^a^	194.94 ± 2.90 ^a^	213.10 ± 6.96 ^a^	232.36 ± 3.17 ^a^	9.53 ± 0.36 ^a^	9.6 ± 0.35 ^a^	10.13 ± 0.32 ^a^
T1	9.57 ± 0.26 ^ab^	9.74 ± 0.28 ^ab^	9.9 ± 0.28 ^ab^	5.02 ± 0.09 ^ab^	5.21 ± 0.08 ^ab^	5.34 ± 0.08 ^b^	189.44 ± 5.34 ^a^	197.69 ± 9.97 ^a^	224.73 ± 4.66 ^ab^	9.47 ± 0.26 ^a^	9.53 ± 0.27 ^a^	10.06 ± 0.26 ^a^
T2	9.37 ± 0.18 ^abc^	9.52 ± 0.17 ^abc^	9.68 ± 0.18 ^abc^	4.94 ± 0.08 ^ab^	5.04 ± 0.09 ^abc^	5.12 ± 0.09 ^bc^	178.90 ± 3.51 ^a^	191.23 ± 4.38 ^a^	201.39 ± 7.70 ^bc^	8.93 ± 0.21 ^a^	9.33 ± 0.33 ^ab^	9.47 ± 0.24 ^ab^
T3	8.89 ± 0.29 ^bc^	9.08 ± 0.29 ^bc^	9.23 ± 0.29 ^bc^	4.76 ± 0.08 ^bc^	4.95 ± 0.07 ^bc^	5.09 ± 0.09 ^bc^	175.32 ± 11.10 ^a^	187.93 ± 8.89 ^ab^	181.09 ± 2.93 ^c^	8.87 ± 0.26 ^a^	9.06 ± 0.18 ^ab^	8.73 ± 0.37 ^bc^
T4	8.8 ± 0.31 ^bc^	9.02 ± 0.31 ^bc^	9.15 ± 0.31 ^bc^	4.73 ± 0.11 ^bc^	4.88 ± 0.12 ^c^	4.98 ± 0.12 ^c^	168.97 ± 3.18 ^a^	155.35 ± 9.12 ^bc^	140.37 ± 9.12 ^d^	8.87 ± 0.27 ^a^	8.6 ± 0.21 ^ab^	8.53 ± 0.22 ^bc^
T5	8.63 ± 0.23 ^c^	8.82 ± 0.212 ^c^	8.94 ± 0.21 ^c^	4.61 ± 0.11 ^c^	4.77 ± 0.11 ^c^	4.89 ± 0.12 ^c^	133.39 ± 2.31 ^b^	120.04 ± 2.40 ^d^	120.65 ± 4.02 ^c^	8.86 ± 0.25 ^a^	8.2 ± 0.33 ^c^	8.07 ± 0.30 ^c^

CK: normal irrigation, spontaneously once in 3 days; T1: irrigating once in 4 days; T2: irrigating once in 5 days; T3: irrigating once in 6 days; T4: irrigating once in 7 days; T5: irrigating once in 8 days. D: days; d: significance of difference. Values for plant height, stem thickness, and number of leaves indicate mean ± SE (*n* = 15), and values for leaf area indicate mean ± SE (*n* = 3). The significance level is *p* < 0.05. Different lowercase letters represent the degree of difference between various treatments applied to the same fruit.

**Table 3:** Influence of water deficit on appearance and morphology of tomato fruit.

Treatments	Equatorial Diameter (mm)	Longitudinal Diameter (mm)	Fruit Shape Index	Fresh Weight of Single Fruit (g)	Single Fruit Dry Weight (g)	Water Content (%)
CK	24.15 ± 0.73 ^a^	19.86 ± 0.88 ^a^	0.82 ± 0.05 ^ab^	4.66 ± 0.26 ^a^	0.61 ± 0.01 ^a^	87 ± 0. 4 ^a^
T1	24.27 ± 1.52 ^a^	19.55 ± 0.17 ^a^	0.81 ± 0.05 ^b^	4.17 ± 0.34 ^a^	0.54 ± 0.03 ^abc^	87 ± 0. 9 ^a^
T2	24.37 ± 0.21 ^a^	19.46 ± 0.32 ^a^	0.80 ± 0.01 ^b^	3.82 ± 0.37 ^ab^	0.50 ± 0.01 ^abc^	86 ± 1.1 ^a^
T3	23.09 ± 0.44 ^a^	19.73 ± 0.25 ^a^	0.85 ± 0.01 ^ab^	3.75 ± 0.20 ^ab^	0.57 ± 0.04 ^ab^	85 ± 0.4 ^ab^
T4	20.46 ± 0.63 ^b^	19.02 ± 0.53 ^ab^	0.93 ± 0.04 ^a^	2.82 ± 0.27 ^bc^	0.47 ± 0.03 ^bc^	83 ± 1.2 ^b^
T5	19.86 ± 0.94 ^b^	17.45 ± 0.63 ^b^	0.88 ± 0.01 ^ab^	2.51 ± 0.44 ^c^	0.43 ± 0.03 ^c^	82 ± 1.3 ^b^

CK: normal irrigation, spontaneously once in 3 days; T1: irrigating once in 4 days; T2: irrigating once in 5 days; T3: irrigating once in 6 days; T4: irrigating once in 7 days; T5: irrigating once in 8 days. Values indicate a mean ± SE (*n* = 3), and the significance level is *p* < 0.05. Different lowercase letters indicate the difference level between different treatments in the same fruit.

## Data Availability

The original contributions presented in this study are included in the article. Further inquiries can be directed to the corresponding author.
